# Role of Chinese Acupuncture in the Treatment for Chemotherapy-Induced Cognitive Impairment in Older Patients With Cancer: Protocol for a Randomized Controlled Trial

**DOI:** 10.2196/53853

**Published:** 2024-02-08

**Authors:** Sunyan Zhao, Jing Zhang, Haijun Wan, Chenjie Tao, Meng Hu, Wei Liang, Zhi Xu, Bingguo Xu, Jiaying Zhang, Guoxin Wang, Ping Li, Guangmei Lyu, Yongling Gong

**Affiliations:** 1 Department of Oncology Nanjing First Hospital Nanjing Medical University Nanjing China; 2 Department of Gastroenterology Jinling Hospital Affiliated Hospital of Medical School, Nanjing University Nanjing China; 3 Department of Oncology Eastern Hepatobiliary Hospital Naval Medical University Shanghai China; 4 Department of Medical Oncology Liyang People’s Hospital Liyang China; 5 Medical Affairs ICON Public Limited Company (ICON Plc) Beijing China; 6 Division of Chinese Medicine Nanjing First Hospital Nanjing Medical University Nanjing China; 7 Department of Gastroenterology Changhai Hospital Naval Medical University Shanghai China; 8 Information Centre Jiangsu Health Vocational College Nanjing China

**Keywords:** older patients with cancer, cognitive impairment, chemobrain, Chinese medicine, electroacupuncture

## Abstract

**Background:**

Older patients with cancer experience cognitive impairment and a series of neurocognitive symptoms known as chemobrain due to chemotherapy. Moreover, older populations are disproportionately affected by chemobrain and heightened negative mental health outcomes after cytotoxic chemical drug therapy. Chinese acupuncture is an emerging therapeutic option for chemotherapy-induced cognitive impairment in older patients with cancer, despite limited supporting evidence.

**Objective:**

Our study aims to directly contribute to the existing knowledge of this novel Chinese medicine mode in older patients with cancer enrolled at the Department of Oncology/Chinese Medicine, Nanjing First Hospital, China, thereby establishing the basis for further research.

**Methods:**

This study involves a 2-arm, prospective, randomized, assessor-blinded clinical trial in older patients with cancer experiencing chemobrain-related stress and treated with Chinese acupuncture from September 30, 2023, to December 31, 2025. We will enroll 168 older patients with cancer with clinically confirmed chemobrain. These participants will be recruited through screening by oncologists for Chinese acupuncture therapy and evaluation. Electroacupuncture will be performed by a registered practitioner of Chinese medicine. The electroacupuncture intervention will take about 30 minutes every session (2 sessions per week over 8 weeks). For the experimental group, the acupuncture points are mainly on the head, limbs, and abdomen, with a total of 6 pairs of electrically charged needles on the head, while for the control group, the acupuncture points are mainly on the head and limbs, with only 1 pair of electrically charged needles on the head.

**Results:**

Eligible participants will be randomized to the control group or the experimental group in 1:1 ratio. The primary outcome of this intervention will be the scores of the Montreal Cognitive Assessment. The secondary outcomes, that is, attentional function and working memory will be determined by the Digit Span Test scores. The quality of life of the patients and multiple functional assessments will also be evaluated. These outcomes will be measured at 2, 4, 6, and 8 weeks after the randomization.

**Conclusions:**

This efficacy trial will explore whether Chinese electroacupuncture can prevent chemobrain, alleviate the related symptoms, and improve the quality of life of older patients with cancer who are undergoing or are just going to begin chemotherapy. The safety of this electroacupuncture intervention for such patients will also be evaluated. Data from this study will be used to promote electroacupuncture application in patients undergoing chemotherapy and support the design of further real-world studies.

**Trial Registration:**

ClinicalTrials.gov NCT05876988; https://clinicaltrials.gov/ct2/show/NCT05876988

**International Registered Report Identifier (IRRID):**

DERR1-10.2196/53853

## Introduction

With the development of new cytotoxic drugs for clinical practice, the clinical curative effects and survival rates of patients with cancer have improved greatly [1]. However, these drugs have considerable adverse side effects that are pernicious, unexpected, and occur at standard doses when administered to older patients with cancer [2]. Studies have shown that older patients with cancer undergoing chemotherapy develop chemotherapy-induced cognitive impairment, known as chemobrain, including a series of neurocognitive symptoms (forgetfulness, trouble concentrating and remembering details, difficulty with multitasking word finding, and taking longer to finish tasks), hospitalizations, and poor quality of life (QoL) [3,4]. In fact, chemobrain-related stress has been reported disproportionately among older individuals with cancer who have undergone chemotherapy [5].

With advances in cancer treatment over the decades, the survival time of older patients with cancer has been gradually extended, and the accompanying symptoms of chemobrain have gradually drawn increasing attention [5]. Recent data have shown that chemobrain-related stress could persist for a long-term period after a systemic therapy, which is particularly relevant to older patients with cancer [6]. These individuals report chemobrain symptoms of anxiety and depressive disorder, and the number of older patients with cancer will increase dramatically in the coming future. Although data on cognitive impairment onset after chemotherapy are lacking for older patients with cancer in China, clinical practitioners have paid constant attention to the health of their constituents, given their propensity for worse physical and mental health outcomes related to chemotherapy [7]. Data suggest that up to two-thirds of older patients with cancer develop chemobrain during or after chemotherapy [8]. Most of these patients experience cognitive impairments for months, but 10%-20% of these individuals experience persistent chemobrain-related stress even after many years of completing chemotherapy. In recent years, the incidence rate of cancer in senile patients has been rising in China [9]. The problem of chemotherapy-induced cognitive dysfunction is becoming increasingly common, and there are growing concerns that chemobrain could become a threat. Therefore, it is crucial to explore an intervention that can effectively prevent and treat chemotherapy-induced cognitive impairment in older patients with tumors.

Pre-existing studies [10] have shown that some neuroprotective factors such as methylphenidate and modafinil are effective for treating chemotherapy-related cognitive impairment; however, most clinical trials on interventions for chemobrain report less access to control groups and less treatment engagement. Combo acupoint stimulation therapy is a new technique based on the theory of traditional Chinese medicine and modern biological principles. With the help of invasive or noninvasive interventions, different stimuli or drug intakes would be administered at acupuncture points, meridians, or related specific body surface sites [11].

Acupuncture has been used for thousands of years in China and other Asian countries to treat various diseases, including sleep disturbance [12]. It is a nonpharmacological therapy that involves inserting needles into acupuncture points and sometimes applying minielectrical current stimulation on acupuncture points via needles or applying acupressure on the surface of points in different parts of the body, including ear and scalp [12]. Electroacupuncture (EA) is an effective family member of combo acupoint stimulation [13]. Lyu et al [14] reported that EA could be effective in alleviating various side effects caused by anticancer drugs, such as pain, vomiting, fever, fatigue, dry mouth, anxiety, depression, and insomnia. In addition, EA could promote the rehabilitation of pathological microstructures in the brain and improve the cognitive ability of patients with cognitive impairment. Clinical trials [14,15] of acupuncture for preventing and managing mild cognitive impairment in older adults show that it can improve the clinical efficacy rate, Mini-Mental State Examination Scale score, Montreal Cognitive Assessment (MoCA) test score, and clock drawing task scores, suggesting that acupuncture can be an effective approach complementary to existing therapies. Evidences [14] from acupuncture in patients after an ischemic stroke support its role in the treatment of neurological deficits through modulation of neuroplasticity. Chemobrain affects cognitive function, in particular, think and memory functions through various mechanisms, including inflammation and oxidative stress, which may lead to neurogenesis and glycogenesis reduction. This pathological process shares some similarities with brain damage after an ischemic stroke [3,16]. Therefore, it is important to investigate if EA is effective in preventing and treating chemobrain in older patients with cancer and for improving the QoL of cancer survivors.

Emerging chemotherapy-induced cognitive impairments in older patients with cancer are likely to widen without proper early intervention tactics. In this protocol, we propose a 2-arm, prospective, randomized, assessor-blinded trial to examine the efficacy and safety of EA for chemobrain among older patients with cancer who are on or about to receive chemotherapy. The aim of this study directly contributes to the existing knowledge in this area, thereby establishing a basis for further research. Changes in the scores on MoCA [17] and the incidence of adverse events serve as the primary outcome. Scores on the Digit Span Test will be the secondary outcome for attentional function and working memory [18]. The QoL and multiple functional assessments will also be evaluated [19]. Outcomes include the MoCA, Digit Span Test, QoL, and multiple functional assessments at 2, 4, 6, and 8 weeks after randomization. Data from this efficacy trial will determine whether Chinese EA successfully improves the symptoms of chemobrain and whether these improvements could markedly reduce the various side effects caused by cytotoxic chemotherapy drugs. If successful, findings from this study might have beneﬁts in reducing chemotherapy-induced working memory impairment. Data from this study may be used to support an implementation and dissemination trial of Chinese EA within real-world behavioral health and social service settings.

## Methods

### Ethics Approval

Eligible participants should provide written informed consent after reviewing consent documents with medical staff. To protect participant privacy and confidentiality, questionnaire surveys for the evaluation of cognitive function should be completed by research staff in a secure office in the inpatient ward. Only trained research staff have access to the key that can match participant data to the participant’s name. This trial has been approved by the ethics committee of Nanjing First Hospital (approval KY20230310-05-KS-0I), and the collaborator of Jiangsu Health Vocational College provides ongoing oversight. This study was registered in ClinicalTrials.gov (NCT05876988) on April 13, 2023, before the first participant was recruited.

### Study Design

**Figure 1:**
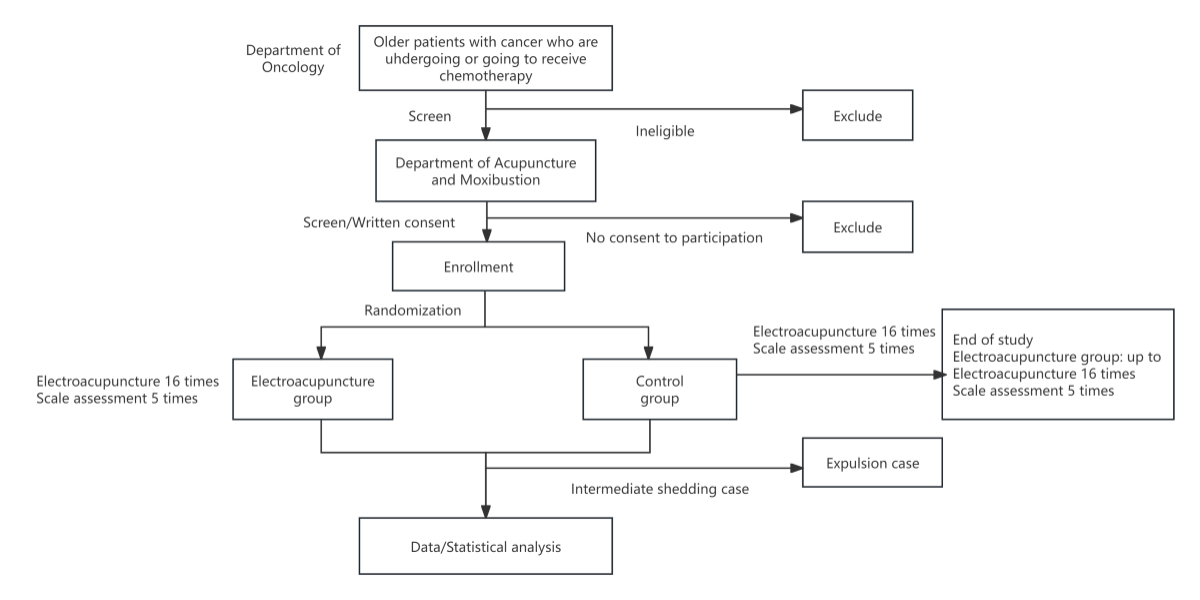
Participant flow diagram in the clinical trial.

We present a protocol for a 2-arm, prospective, randomized, assessor-blinded trial to examine the efficacy and safety of EA for chemobrain among older patients with cancer who are undergoing chemotherapy or those are going to just begin chemotherapy. This study refers to a double-blinded trial in which neither the investigator nor the participant knows the group to which the participant belongs (experimental group or control group), and the analyst usually does not know which group the data being analyzed belong to. This study is designed to eliminate subjective biases and personal preferences that may arise in the consciousness of investigators and participants. Those who report clinically significant cognitive impairment are being recruited and enrolled to participate in a trial on the effects of a novel EA intervention to address negative mood symptoms and reduce chemotherapy-induced adverse reactions. Participants are recruited via community organizations, social media, and internet outlets (eg, hospital classified ads, WeChat). Interested older patients with cancer complete a screener assessment by the medical staff of the Oncology department or a Chinese medicine doctor, and they report the levels of cognitive dysfunction they are experiencing through the Overall Anxiety Severity and Impairment Scale [20] and the Overall Depression Severity and Impairment Scale [21]. Those eligible at the screener complete an enrollment for full eligibility and receive instructions on how to accept EA therapy to complete the baseline assessment. Additionally, eligible participants will be randomly assigned to either an experimental group or a control group at 1:1 ratio. Participants then engage with the assigned intervention for 8 weeks following randomization. Participants complete follow-up assessments at baseline, 2, 4, 6, and 8 weeks after randomization, which include the MoCA, Digit Span Test, QoL, and multiple functional assessments. Additionally, participants are prompted to complete ecological momentary assessments [22] twice weekly throughout the 2-month postrandomization period. The study flow diagram is shown in Figure 1.

### Specific Aims and Hypotheses

Chinese acupuncture is an emerging therapeutic option for chemotherapy-induced cognitive impairment in older patients with cancer, despite limited supporting evidence [23]. This study has 2 specific aims.

1. First, to evaluate the clinical therapeutic response of EA intervention for the treatment of chemobrain in older patients with cancer, a randomized controlled trial is designed to study the effects of EA on chemobrain at baseline, 2, 4, 6, and 8 weeks follow-up. We hypothesize that those assigned to EA will show greater reductions from baseline to follow-ups in MoCA Overall Anxiety Severity and Impairment Scale and Digit Span Test scores and greater reductions in cognitive impairment in daily responsibilities relative to the control group. We also hypothesize that the effectiveness of EA on QoL and multiple functional assessments will be similar across experimental or control groups.
2. Second, our aim is to elucidate the modern biomedical mechanism of EA in the treatment of chemobrain in older patients with cancer. In testing the putative mechanisms of action, we hypothesize that the intervention effects on study outcomes will be mediated by reductions in chemotherapy-induced working memory impairment and the incidence of certain digestive, neurological, and distress-related symptoms.

### Participants and Recruitment

**Figure 2:**
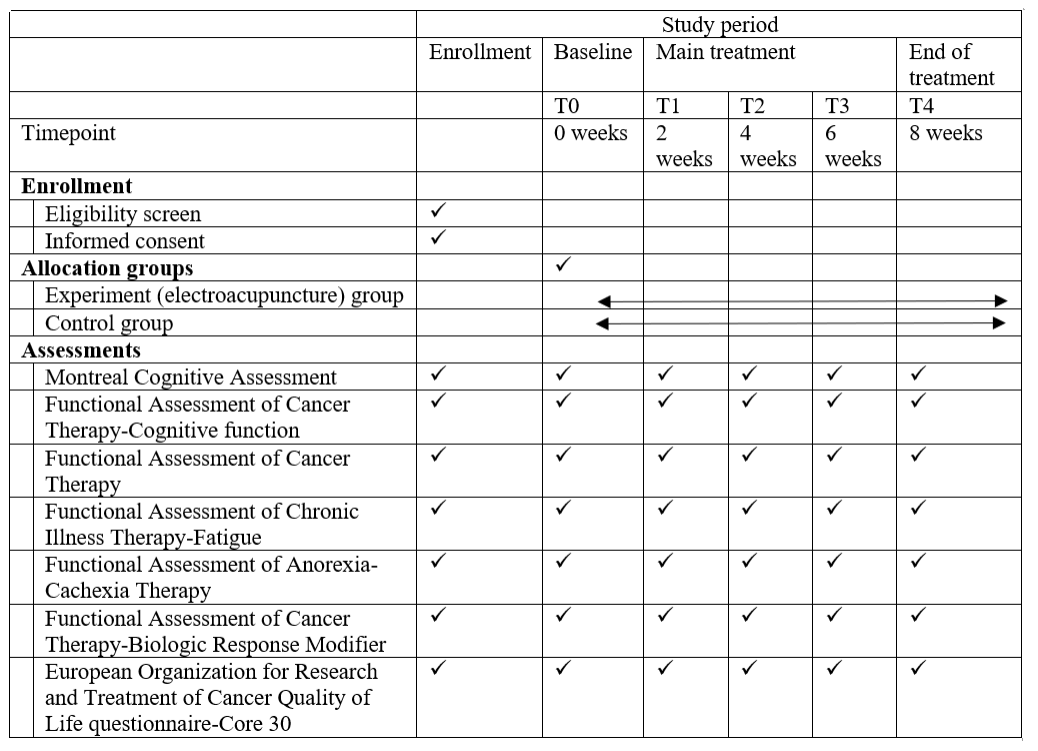
Schedule of enrollment, interventions, and assessments. T0: baseline; T1-T3: midpoint of main treatment once every 2 weeks; T4: end of the 8-week main treatment.

This 2-arm, prospective, randomized, assessor-blinded trial will be conducted from September 2023 to December 2025 in the Department of Oncology/Chinese Medicine, Nanjing First Hospital, China. Potentially eligible participants would be screened and recruited through clinical oncologists/Chinese medicine acupuncturists’ referral from local hospitals and advertisements. Enrollment to this study began in September 2023 and, as of writing this paper, is in the recruitment phase. Enrollment, randomization, intervention delivery, and assessments are completed by trained research staff. Both study conditions receive the same set of questionnaires at each assessment (see Figure 2) or the full list and timeline of study measures. These questionnaires assess chemobrain symptoms, functional impairment, general and chemotherapy-specific affect constructs, and sociocultural factors. Interested individuals complete an initial screener to assess age, racial/ethnic identity, clinical confirmation of chemobrain, state of residence, and willingness to complete study assessments. During the enrollment, volunteer participants will be asked to sign written informed consent forms and are informed of the purpose, goals, and procedures of the study. All participants will give voluntary, written, and informed consent before entering the trial.

### Inclusion and Exclusion Criteria

The participants in this study are 168 older patients with cancer who are further assessed for eligibility and meet the following eligibility criteria: (1) have clinically pathological diagnosis of malignant tumor, (2) aged 60 years or older, and (3) are currently undergoing or just about to commence a comprehensive treatment of chemotherapy. The exclusion criteria are as follows: (1) undergone chemotherapy within the last 2 years; (2) metal device or pacemaker planted in the body, developed epilepsy, and experienced instability; (3) participated in a drug trial within the last 6 months; (4) alcohol or drug abuse history in the past year; and (5) those who are afraid of acupuncture.

### Study Procedures

After screening, eligible participants will be enrolled and they will complete baseline assessments. Thereafter, participants will be randomly assigned to either an EA experimental group or a sham control group. EA or sham treatments will be given twice weekly for 8 weeks—a total of 16 sessions. Participants in both groups will receive routine chemotherapy provided by their oncologists, which will include the use of cytotoxic drugs. Participants will be assessed at the following timepoints (see Figure 2): baseline (T0), midpoint of main treatment (T1-T3) once every 2 weeks, and end of the 8-week main treatment (T4). Assessments include MoCA [24] + Functional Assessment of Cancer Therapy-Cognitive function (FACT-Cog) [25] for cognitive performance, FACT/Functional Assessment of Chronic Illness Therapy-Fatigue (FACIT-Fatigue) [26] for fatigue, European Organization for Research and Treatment of Cancer QoL Questionnaire-Core 30 (EORTC QLQ-C30) [27] for QoL, and Functional Assessment of Anorexia-Cachexia Therapy (FAACT)/FACT-Biologic Response Modifier (FACT-BRM) [28] for chemotherapy-related side effects. Assessments will be done prior to any procedures in that visit, including disease assessment and physician consultation.

### Randomization and Allocation Concealment

Older patients with tumors will be screened for eligibility, and enrollment and baseline assessments should be completed thereafter. A random code of simple, complete, and nonsequential numbers could be produced in advance from a computer-generated block randomization with random block sizes. Participants will be randomly assigned to experimental or control groups in a 1:1 ratio. Random information will be sealed in sequentially numbered opaque envelopes.

### Blinding

Only the acupuncturist can open the envelope after the participant’s completion of baseline assessments. Participants and all other researchers will be blinded to group assignments. Participants are informed that they have a similar chance of allocation to any group and will be blinded to group assignments. A clinical assessor will be arranged to conduct assessments on a day different from the day of acupuncture treatment to avoid cross communications among the assessors, acupuncturists, and participants. An eye mask will be used to avoid communications among participants during EA treatment.

### Interventions

Potentially eligible patients will be recruited through clinical oncologists’ referral from local hospitals and advertisements. Research staff are notified once a participant completes the baseline assessment. The interventions of the experimental and control groups will be performed in the Department of Medical Oncology, Nanjing First Hospital, Nanjing Medical University, Nanjing, Jiangsu, China. Registered Chinese medicine acupuncturists at the Division of Chinese Medicine will be recruited who will be responsible for delivering treatment according to STRICTA (Standards for Reporting Interventions in Clinical Trials of Acupuncture) [29].

### Acupuncture Group

**Figure 3:**
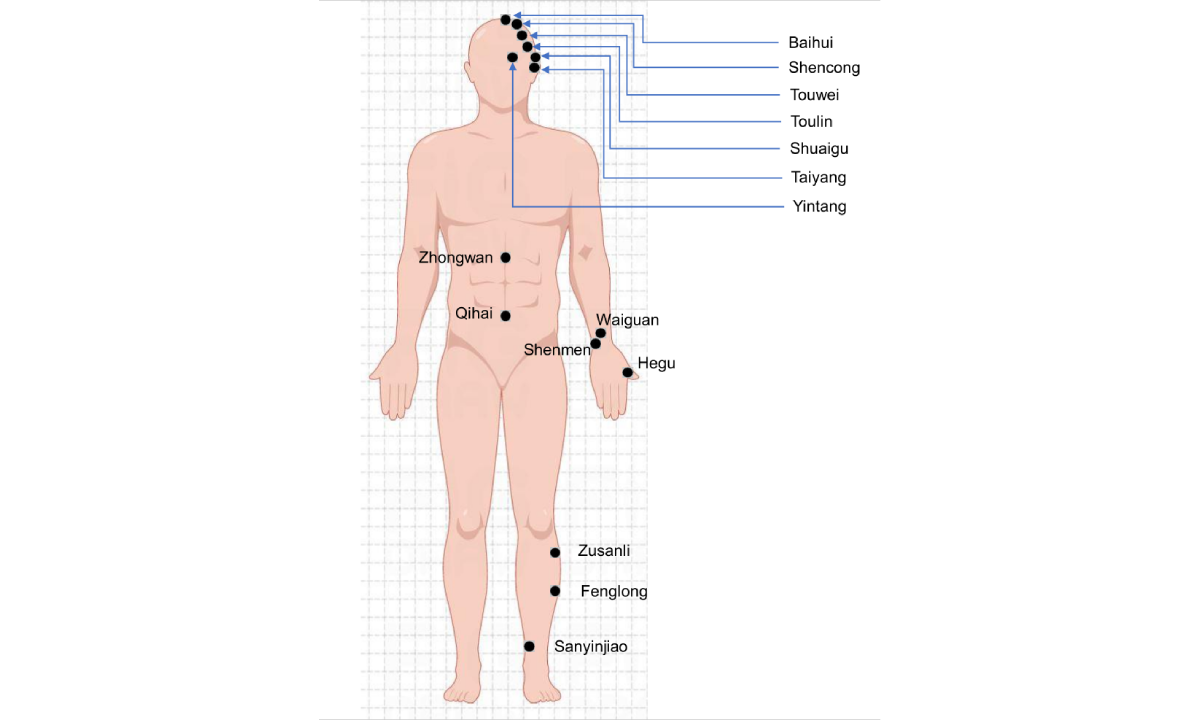
Acupoints for electroacupuncture stimulation.

Participants in the experimental group will receive EA treatment. Acupuncture intervention will be conducted for 2 sessions per week over 8 consecutive weeks. Assessments will be conducted at baseline and once biweekly thereafter. For the experimental group, acupuncture points are mainly on the head, limbs, and abdomen, with a total of 6 pairs of electrically charged needles on the head, while for the control group, acupuncture points are mainly on the head and limbs, with only a pair of electrically charged needles on the head. The acupuncture points are as follows: Baihui point, left Shencong point, right Shencong point, bilateral Shuaigu point, bilateral Touwei point, bilateral Toulin point, bilateral Taiyang point, Yintang point, Zhongwan point, Qihai point, bilateral Waiguan point, bilateral Shenmen point, bilateral Hegu point, bilateral Zusanli point, bilateral Fenglong point and bilateral Sanyinjiao point. A total of 6 pairs of electrodes from the electric stimulator (CMNS6-3; 2-5 Hz, continuous wave) will be connected to the end of the needles. The 6 fixed points are Baihui and Yintang, left Shencong and left Toulin, right Shencong and right Toulin, left Shuaigu and right Shuaigu point, left Touwei and right Touwei points, and left Taiyang and right Taiyang (see Figure 3).

Disposable acupuncture needles (0.30 mm in diameter and 25-40 mm in length) will be inserted at a depth of 10-30 mm perpendicularly or obliquely into the acupoints. Manual manipulation will be performed for all acupoints to evoke needling sensation. Electrical stimulation will be additionally delivered on the 6 pairs of the frontal acupoints. The output peak current and voltage of the machine would be 6 V and 48 mA, respectively, with constant waves at frequencies of 2 Hz and phase duration of 100 µs for 30 minutes. Electrical stimulation will last for 30 minutes. The needles on body acupoints will also be retained for 30 minutes. Upon needle withdrawal, the points will be compressed with iodine volts swabs to prevent bleeding [30].

### Control Group

The control treatment procedure is the same as that in the experimental group, except that only a pair of electric needles will be selected for the electrical stimulation on the head. After completing the 8-week acupuncture intervention, patients in the control group will be administered follow-up EA acupuncture free of charge. The same 16 sessions or a total of no more than 8 weeks of acupuncture will be performed as compensation.

### Assessments

In this study, therapeutic efficiency will be assessed in terms of the following aspects: cognitive performance, fatigue, QoL, and chemotherapy-induced side effects. The primary outcome of this study is to verify whether EA is therapeutically useful for preventing chemobrain and alleviating the related symptoms and the safety of intervention. Cognitive performance will be assessed using MoCA + FACT-Cog. The secondary outcomes are as follows: QoL will be measured using EORTC QLQ-C30, fatigue will be assessed using the FACIT-Fatigue scale, and the manifestation of chemotherapy-induced side effects will be assessed using the Chemotherapy Symptom Rating Scale FAACT + FACT-BRM. All assessment scales will be evaluated by previously trained researchers at the following timepoints (see Figure 2): baseline (T0), midpoint of main treatment (T1-T3) once every 2 weeks, and end of the 8-week main treatment (T4) (a total of 5 timepoints). We will also keep a record of all the shedding cases. This study is a multidisciplinary clinical trial (across oncology and Chinese medicine disciplines) on the role of Chinese acupuncture as therapy for chemotherapy-induced cognitive impairment in older patients with cancer. If successful, findings from this study might have beneﬁts in reducing chemotherapy-induced working memory impairment. Data from this study may be used to support an implementation and dissemination trial of Chinese EA in real-world behavioral health and social service settings.

### Questionnaires/Instruments

#### MoCA Self-Reported Questionnaire

The MoCA self-reported questionnaire is the main method used for assessing cognitive function. To objectively measure the cognitive impairment patterns of the participants, MoCA, a sensitive, time-sparing tool with high diagnostic validity, will be used to measure the degree of chemobrain. We evaluate the utility of MoCA as a chemobrain screening test for older patients with cancer who are undergoing or about to receive chemotherapy. Participants will be instructed to self-report their cognitive functions through MoCA for 5 consecutive timepoints from baseline to the end of the 8-week treatment. Statistical software will be used to analyze participants’ cognitive function information recorded in case report forms.

#### FACT-Cog Questionnaire

FACT-Cog is a subjective measure of chemobrain. The FACT-Cog is a 37-item member of the FACIT suite of questionnaires, which is made up of 4 subscales: perceived cognitive impairments (18 items), perceived cognitive abilities (7 items), impact of perceived cognitive impairment on QoL (4 items), and comments from others on cognitive function (4 items) [24]. FACT-Cog is considered a reliable and valid patient report of chemotherapy-induced cognitive symptoms. Participants are required to report their responses in FACT-Cog just the same as MoCA.

#### FACT Instrument

FACT is a 37-item self-reported instrument devised to assess the multidimensional health-related QoL in patients with cancer. It consists of 5 subscales, namely, physical, social/family, emotional, functional well-being, and additional concerns for cancer. Each item is scored on a 0 to 4 scale, and the sum of all 5 subscales ranges from 0 to 144. A higher score indicates a better QoL [12].

#### FACIT-Fatigue Self-Report Questionnaire

The FACIT-Fatigue is a valid self-reported questionnaire for assessing anemia-related fatigue in patients with cancer. This 13-item questionnaire comprises concepts related to the severity and impact of fatigue over the last 7 days, including varied fatigue-related concepts across its 13 items, allowing for a nuanced assessment.

#### EORTC QLQ-C30 Questionnaire

The EORTC QLQ-C30 will be used as a self-reported questionnaire for interpreting group-level results in older patients with cancer. The current estimates can be used to better interpret the results of clinical trials in older patients with cancer using the EORTC QLQ-C30 questionnaire.

#### FAACT Self-Administered Questionnaire

The FAACT is a self-administered questionnaire developed to assess symptoms in patients with cancer with limited food intake and decreased QoL. For FAACT, a cutoff value of ≤24 has been advised to assess anorexia based on the fact that it is the half of the maximum score than can be obtained [31].

#### FACT-BRM Screening Tool

The FACT-BRM is a brief screening tool designed to assess the QoL in patients treated with BRMs. The FACT-BRM scale will be useful for measuring QoL in older patients with cancer who are receiving treatment with BRMs.

#### Safety Assessment

Except for the sensation of soreness, numbness, swelling, and heaviness, the EA intervention will not usually cause significant discomfort or serious adverse effects, but some participants may experience pain or subcutaneous ecchymosis. Patients who have acupuncture on an empty stomach are more likely to experience dizziness. Subcutaneous ecchymosis will disappear spontaneously if the participant’s blood coagulation is normal. Patients will be reminded to avoid acupuncture on an empty stomach. The condition of each participant will be closely monitored, and if other symptoms or adverse effects occur, the investigator will ensure that the participant is receiving the appropriate treatment and is suitable to continue receiving EA. All participants in the clinical trial will be asked to evaluate the safety of the intervention, and any adverse events experienced during acupuncture treatment will be reported at each visit. The severity of adverse events will be assessed according to the Common Terminology Standard for Adverse Events v5.0 criteria [32], which are useful for determining the causal relationship between acupuncture and adverse reactions. Serious adverse events will be immediately reported to the project investigator and ethics committee. The investigator will also discuss the situation with the participant to plan any temporary discontinuation or withdrawal. The costs will be reimbursed by the investigator’s organization.

#### Sample Size Estimation

A total of 168 older patients with cancer will be recruited, of which 84 will be in the experimental group (EA) and 84 will be in the control group. Sample size calculation:

n = [p1 (1 – p1) + p2 (1 – p2)]/(p1 – p2)] × Cp.power

Cp refers to the process capability index. In the 2-sided test, Cp.power = 10.5 when *P*<.05 is met. n is the number of patients to be recruited in each group, p1 and p2 represent the incidence in each group, and a mean difference with 95% level of significance (α) and 80% power (1 – β) is set. In older patients with cancer, the incidence of chemobrain after chemotherapy is 30%, and the reduction of chemobrain could be 10%-20% with acupuncture intervention. The sample size for each group is 84 participants, considering a 20% dropout. Therefore, the overall target sample size of 168 will be included.

### Statistical Analyses

Cognitive impairment is defined as those with SD>1.5 in the mean scores of MoCA and the QLQ-C30 at baseline for all participants at different timepoints. A time-series test in the Kaplan-Meier survival analysis will be used to compare the incidence of cognitive deficits accumulated after 8 weeks of acupuncture intervention between the 2 groups. Linear mixed-effects modeling will be used to compare participants’ cognitive impairment, fatigue, and depression at baseline, during, and after acupuncture intervention, where only those who have completed at least one assessment at baseline and during treatment will be compared. This model will use time and group as categorical fixed factors and random intercepts and use random intercepts in conjunction with a measurement covariance matrix. Participants’ age, chemotherapeutic drug dosage, fatigue at baseline, depression, and additional treatments will be considered as covariates in the statistical analyses. A 2-sided *t* test will be used to compare the covariates at each timepoint between the 2 groups. The chi-square test will be used for qualitative information and the *t* test will be used for continuous information at baseline. Statistical significance will be considered at *P*<.05. The study data used will be statistically analyzed using SPSS software (IBM Corp) under the supervision of a statistician. To ensure the privacy of the patients, all relevant study data and personal data will be kept in a locked file cabinet. Electronic data will be stored on an encrypted computer and will not be stored on any external storage device. Each participant will be assigned his or her own ID in the study record, which will be used in lieu of his or her name and personal identifying information. Clinical data from the study will be kept in a separate file from the participant’s personal data. The principal investigator will be responsible for all the clinical trial data. During and after the clinical trial, the principal investigator, researchers, and the ethical review committee have the right to view the study data. Participants will have the right to access their personal data and the results of the study. All study data will be stored for a minimum of 3 years after the completion of the trial, after which they will be destroyed in accordance with the rules of the ethics review committee.

## Results

This clinical trial was funded by the Jiangsu Health Vocational College and Nanjing First Hospital in January 2023. The ethics review committee approval was finalized in April 2023, and data collection began on September 1, 2023. Investigators have since been engaged in activities associated with study enrollment and data collection, including recruitment, screening, consenting, and enrolling participants; administering EA intervention; timepoint assessing; and monitoring adverse events and resolving problems early. As of October 21, 2023, 11 participants have been recruited into the clinical trial. A total of 168 older patients with cancer who are undergoing or are just about to commence chemotherapy will be enrolled in this clinical trial, and they will be randomly assigned into either the experimental group or the control group of 84 participants each. A total of 16 EA treatments (twice per week) will be administered to each participant for 8 weeks, and the participants will be assessed for cognitive performance, fatigue, and QoL. Concurrently, they will receive conventional chemotherapy. Improvement or even relief of chemobrain symptoms in older patients with cancer undergoing chemotherapy will be expected if the results of the EA intervention are satisfactory.

## Discussion

Patients with cancer may have concerns of psychiatric and digestive symptoms while undergoing or just after having recently undergone chemotherapy [3]. The most common clinical manifestations are fatigue, nausea, vomiting, dry mouth, anxiety, depression, and insomnia. More seriously, cognitive impairments with a range of neurocognitive symptoms occur frequently, including visual and verbal memory impairment, forgetfulness, difficulty concentrating, learning difficulties, and organizational and coordination dysfunction [3,4]. Since the 1980s, researchers have been studying chemotherapy-induced cognitive impairment and have given several definitions to this condition, such as postchemotherapy cognitive impairment and chemotherapy-induced cognitive dysfunction. Recently, this condition was termed as chemobrain [3-5].

Chemobrain is a frequent side effect experienced by an increasing number of older patients with cancer, and it has significantly impacted their QoL [4]. Chemobrain can be described as cognitive symptoms reported by patients with cancer in self-reported questionnaires or as cognitive changes evaluated by formal neuropsychological tests [5]. Older patients with cancer will experience cognitive impairment due to chemoradiotherapy, which will lead to a series of neurocognitive symptoms called chemobrain. Older populations have been disproportionately affected by chemobrain and have heightened negative mental health outcomes after cytotoxic chemical drug therapy [3,5,7].

According to available literature [3,14], more than 75% of patients with cancer experience cognitive impairment while undergoing chemotherapy or just after completing chemotherapy, with 15%-30% of these patients experiencing persistent symptoms for up to decades. The prevalence of postchemotherapy cognitive impairment is as high as 50%-75% among older cancer survivors [33]. In some circumstances, the cognitive impairment can be lifelong, affecting the patient’s mood, relationships, QoL, career, social activity, and family life. Considering the increasing incidence of cancer, chemobrain is quite complicated, increasingly important, and has far-reaching consequences. As the incidence of cancer survivors rises, the adverse effects of chemobrain will become more frequent, thus necessitating the need to effectively prevent and treat chemobrain.

At present, there is no responsive therapy for chemobrain. The current clinical medications are not effective for chemobrain prevention and therapy. Study and research literatures [14,15] suggest that Chinese acupuncture might be effective for relieving various discomforts caused by anticancer drugs, such as pain, vomiting, fever, fatigue, dry mouth, anxiety, depression, and sleep disorders. Several studies [11,13-16,30] have shown the efficacy of EA intervention in various neurological diseases. Compared with acupuncture, EA has reported to produce a higher intensity of stimulation on acupoints and is progressively being widely used for its adjustable strength, frequency, and easy quantification in clinical practice [14]. In addition, EA can promote the rehabilitation of pathological microstructures in the brain and improve the cognitive function of patients with cognitive impairment [16].

Our study aims to explore the feasibility, efficacy, and safety of using EA by involving multiple disciplines (oncology and Chinese Medicine) to treat chemobrain in older patients with cancer. Acupuncture itself has been demonstrated as a safe and effective treatment, and EA is a widely accepted and cost-effective alternative therapy option with low adverse effects for older patients with cancer. The findings of this study will provide useful information on determining a suitable therapy for chemobrain in older patients with cancer. EA may be ready for implementation and dissemination in real-world cognitive function settings consistent with the objectives outlined in this clinical trial’s strategic plan. Overall, this proposal has the potential to decrease neurocognitive symptoms among older patients with cancer—populations determined to be most at risk of exacerbated, long-lasting negative health sequelae. EA, if proven to be effective, can be implemented into routine settings to benefit older patients with cancer experiencing chemobrain. We believe that data from this efficacy trial will determine whether Chinese EA successfully alleviates the symptoms of chemobrain and whether these improvements could markedly reduce the various side effects due to cytotoxic chemical drugs.

In conclusion, the goal of this clinical trial is to target and reduce emerging and likely exacerbated cognitive impairments caused by chemotherapy among older patients with cancer. We aim to evaluate this novel Chinese medicine mode of EA in older patients with cancer by developing a therapeutic responsive treatment for chemobrain. By addressing neurocognitive symptoms through an EA intervention that targets underlying pathophysiological and psychological factors, we hope that this EA intervention will reduce the symptoms of chemobrain in older patients with cancer. If successful, findings from this study might have beneﬁts in reducing chemotherapy-induced working memory impairment. Data from this study may be used to support an implementation and dissemination trial of Chinese EA within real-world behavioral health and social service settings.

## Abbreviations

EA: electroacupuncture
EORTC QLQ-C30: European Organization for Research and Treatment of Cancer Quality of Life Questionnaire-Core 30
FAACT: Functional Assessment of Anorexia-Cachexia Therapy
FACIT-Fatigue: Functional Assessment of Chronic Illness Therapy-Fatigue
FACT: Functional Assessment of Cancer Therapy
FACT-BRM: Functional Assessment of Cancer Therapy-Biologic Response Modifier
FACT-Cog: Functional Assessment of Cancer Therapy-Cognitive function
MoCA: Montreal Cognitive Assessment
QoL: quality of life
STRICTA: Standards for Reporting Interventions in Clinical Trials of Acupuncture

## Data Availability

The data sets generated and analyzed during this study will be available from the corresponding author on reasonable request following the completion of the trial and publication of the main outcomes paper. Deidentified data also will be made available via the Jiangsu Health Vocational College and Nanjing First Hospital.

## References

[1] Luo Z, He Y, Ma G, Deng Y, Chen Y, Zhou Y, Xu X, Li X, Du Y (2020). Years of life lost due to premature death and their trends in people with malignant neoplasm of female genital organs in Shanghai, China during 1995-2018: a population based study. BMC Public Health.

[2] Sienkiewicz K, Burzyńska M, Rydlewska-Liszkowska I, Sienkiewicz J, Gaszyńska E (2021). The importance of direct patient reporting of adverse drug reactions in the safety monitoring process. Int J Environ Res Public Health.

[3] Lv L, Mao S, Dong H, Hu P, Dong R (2020). Pathogenesis, assessments, and management of chemotherapy-related cognitive impairment (CRCI): an updated literature review. J Oncol.

[4] Vega JN, Dumas J, Newhouse PA (2017). Cognitive effects of chemotherapy and cancer-related treatments in older adults. Am J Geriatr Psychiatry.

[5] Walczak P, Janowski M (2019). Chemobrain as a product of growing success in chemotherapy - focus on glia as both a victim and a cure. Neuropsychiatry (London).

[6] Was H, Borkowska A, Bagues A, Tu L, Liu JYH, Lu Z, Rudd JA, Nurgali K, Abalo R (2022). Mechanisms of chemotherapy-induced neurotoxicity. Front Pharmacol.

[7] Piechotta V, Adams A, Haque M, Scheckel B, Kreuzberger N, Monsef I, Jordan K, Kuhr K, Skoetz N (2021). Antiemetics for adults for prevention of nausea and vomiting caused by moderately or highly emetogenic chemotherapy: a network meta-analysis. Cochrane Database Syst Rev.

[8] Sekeres MJ, Bradley-Garcia M, Martinez-Canabal A, Winocur G (2021). Chemotherapy-induced cognitive impairment and hippocampal neurogenesis: a review of physiological mechanisms and interventions. Int J Mol Sci.

[9] Duan W, Zheng A, Mu X, Li M, Liu C, Huang W, Wang X (2017). How great is the medical burden of disease on the aged? Research based on "System of Health Account 2011". Health Qual Life Outcomes.

[10] Balasubramanian P, Kiss T, Tarantini S, Nyúl-Tóth Ádám, Ahire C, Yabluchanskiy A, Csipo T, Lipecz A, Tabak A, Institoris A, Csiszar A, Ungvari Z (2021). Obesity-induced cognitive impairment in older adults: a microvascular perspective. Am J Physiol Heart Circ Physiol.

[11] Ni X, Dong L, Tian T, Liu L, Li X, Li F, Zhao L (2020). Acupuncture versus various control treatments in the treatment of migraine: a review of randomized controlled trials from the past 10 years. J Pain Res.

[12] Zhang J, Yang M, So TH, Chang TY, Qin Z, Chen H, Lam WL, Yeung WF, Chung KF, Jiang F, Lao L, Zhang Z (2021). Electroacupuncture plus auricular acupressure on chemotherapy-related insomnia in patients with breast cancer (EACRI): study protocol for a randomized, sham-controlled trial. Integr Cancer Ther.

[13] Jiang X, Tian Y, Xu L, Zhang Q, Wan Y, Qi X, Li B, Guo J, Sun W, Luo A, Huang J, Gu X (2019). Inhibition of triple-negative breast cancer tumor growth by electroacupuncture with encircled needling and its mechanisms in a mice xenograft model. Int J Med Sci.

[14] Lyu YR, Lee H, Park H, Kwon O, Kim A, Jung IC, Park Y, Cho J, Kim J, Kim M, Lee J, Kim J (2022). Electroacupuncture for cancer-related cognitive impairment: a clinical feasibility study. Integr Cancer Ther.

[15] Zhang Zhang-Jin, Man Sui-Cheung, Yam Lo-Lo, Yiu Chui Ying, Leung Roland Ching-Yu, Qin Zong-Shi, Chan Kit-Wa Sherry, Lee Victor Ho Fun, Kwong Ava, Yeung Wing-Fai, So Winnie KW, Ho Lai Ming, Dong Ying-Ying (2020). Electroacupuncture trigeminal nerve stimulation plus body acupuncture for chemotherapy-induced cognitive impairment in breast cancer patients: An assessor-participant blinded, randomized controlled trial. Brain Behav Immun.

[16] Chang Qwang-Yuen, Lin Yi-Wen, Hsieh Ching-Liang (2018). Acupuncture and neuroregeneration in ischemic stroke. Neural Regen Res.

[17] Pirani A, Nasreddine Z, Neviani F, Fabbo A, Rocchi MB, Bertolotti M, Tulipani C, Galassi M, Belvederi Murri M, Neri M (2022). MoCA 7.1: Multicenter validation of the first Italian version of Montreal Cognitive Assessment. J Alzheimers Dis Rep.

[18] On AK, Hwang K, Jang S, Lee H, Soh M, Yang C, Lee S (2020). Detection of malingering using Wechsler Adult Intelligence Scale-IV for psychiatric patients. Psychiatry Investig.

[19] Suica Z, Behrendt F, Gäumann Szabina, Gerth U, Schmidt-Trucksäss Arno, Ettlin T, Schuster-Amft C (2022). Imagery ability assessments: a cross-disciplinary systematic review and quality evaluation of psychometric properties. BMC Med.

[20] Hiller TS, Hoffmann S, Teismann T, Lukaschek K, Gensichen J (2023). Psychometric evaluation and Rasch analyses of the German Overall Anxiety Severity and Impairment Scale (OASIS-D). Sci Rep.

[21] Health Quality Ontario (2017). Psychotherapy for major depressive disorder and generalized anxiety disorder: a health technology assessment. Ont Health Technol Assess Ser.

[22] Bittel KM, O'Briant KY, Ragaglia RM, Buseth L, Murtha C, Yu J, Stanely JM, Hudgins BL, Hevel DJ, Maher JP (2023). Associations between social cognitive determinants and movement-related behaviors in studies using ecological momentary assessment methods: systematic review. JMIR Mhealth Uhealth.

[23] Wang S, Lin H, Cong W (2019). Chinese medicines improve perimenopausal symptoms induced by surgery, chemoradiotherapy, or endocrine treatment for breast cancer. Front Pharmacol.

[24] Khan G, Mirza N, Waheed W (2022). Developing guidelines for the translation and cultural adaptation of the Montreal Cognitive Assessment: scoping review and qualitative synthesis. BJPsych Open.

[25] Bell ML, Dhillon HM, Bray VJ, Vardy JL (2018). Important differences and meaningful changes for the Functional Assessment of Cancer Therapy-Cognitive Function (FACT-Cog). J Patient Rep Outcomes.

[26] Cai T, Chen J, Ni F, Zhu R, Wu F, Huang Q, Zhou T, Yang Y, Yuan C (2023). Psychometric properties of the Chinese version of the functional assessment of chronic illness therapy-fatigue (FACIT-F) among patients with breast cancer. Health Qual Life Outcomes.

[27] Imran M, Al-Wassia R, Alkhayyat SS, Baig M, Al-Saati BA (2019). Assessment of quality of life (QoL) in breast cancer patients by using EORTC QLQ-C30 and BR-23 questionnaires: A tertiary care center survey in the western region of Saudi Arabia. PLoS One.

[28] Blauwhoff-Buskermolen S, Ruijgrok C, Ostelo RW, de Vet HCW, Verheul HMW, de van der Schueren MAE, Langius JAE (2016). The assessment of anorexia in patients with cancer: cut-off values for the FAACT-A/CS and the VAS for appetite. Support Care Cancer.

[29] MacPherson H, Altman DG, Hammerschlag R, Youping L, Taixiang W, White A, Moher D, STRICTA Revision Group (2010). Revised STandards for Reporting Interventions in Clinical Trials of Acupuncture (STRICTA): extending the CONSORT statement. PLoS Med.

[30] Zhang J, Qin Z, So TH, Chang TY, Yang S, Chen H, Yeung WF, Chung KF, Chan PY, Huang Y, Xu S, Chiang CY, Lao L, Zhang Z (2023). Acupuncture for chemotherapy-associated insomnia in breast cancer patients: an assessor-participant blinded, randomized, sham-controlled trial. Breast Cancer Res.

[31] Muscaritoli M, Anker S, Argilés J, Aversa Z, Bauer J, Biolo G, Boirie Y, Bosaeus I, Cederholm T, Costelli P, Fearon K, Laviano A, Maggio M, Rossi Fanelli F, Schneider S, Schols A, Sieber C (2010). Consensus definition of sarcopenia, cachexia and pre-cachexia: joint document elaborated by Special Interest Groups (SIG) "cachexia-anorexia in chronic wasting diseases" and "nutrition in geriatrics". Clin Nutr.

[32] U.S. DOHS (2017). Common terminology criteria for adverse events (CTCAE) v5. 0 Nov.

[33] Országhová Zuzana, Mego M, Chovanec M (2021). Long-term cognitive dysfunction in cancer survivors. Front Mol Biosci.

